# Architectural organization and molecular profiling of 3D cancer heterospheroids and their application in drug testing

**DOI:** 10.3389/fonc.2024.1386097

**Published:** 2024-07-01

**Authors:** Boye Schnack Nielsen, Natasha Helleberg Madsen, Jesper Larsen, Isabella Skandorff, Monika Gad, Kim Holmstrøm

**Affiliations:** Department of Cellular Engineering & Disease Modeling, Bioneer A/S, Hørsholm, Denmark

**Keywords:** 3D cell culture, drug testing, gene expression profiling, fibroblasts, heterospheroids, hypoxia

## Abstract

3D cancer cell cultures have enabled new opportunities for replacing compound testing in experimental animals. However, most solid tumors are composed of multiple cell types, including fibroblasts. In this study we developed multicellular tumor heterospheroids composed of cancer and fibroblasts cell lines. We developed heterospheroids by combining HT-29, MCF-7, PANC-1 or SW480 with 1BR.3.G fibroblasts, which we have previously reported support spheroid formation. We also tested fibroblast cell lines, MRC-5, GM00498 and HIF, but 1BR.3.G was found to best form heterospheroids with morphological similarity to *in vivo* tumor tissue. The architectural organization of heterospheroids was based on histological examination using immunohistochemistry. We found that HT-29 and MCF-7 cells developed spheroids with the cancer cells surrounding the fibroblasts, whereas PANC-1 cells interspersed with the fibroblasts and SW480 cells were surrounded by fibroblasts. The fibroblasts also expressed collagen-1 and FAP-α, and whole transcriptomic analysis (WTA) showed abundant ECM- and EMT-related expression in heterospheroids, thus reflecting a representative tumor-like microenvironment. The WTA showed that PANC-1 heterospheroids possess a strong EMT profile with abundant Vimentin and CDH2 expression. Drug testing was evaluated by measuring cytotoxicity of 5FU and cisplatin using cell viability and apoptosis assays. We found no major impact on the cytotoxicity when fibroblasts were added to the spheroids. We conclude that the cancer cell lines together with fibroblasts shape the architectural organization of heterospheroids to form tumor-like morphology, and we propose that the various 3D tumor structures can be used for drug testing directed against the cancer cells as well as the fibroblasts.

## 1 Introduction

Malignant solid tumors are typically formed around epithelial cancer cells that orchestrate a tumor stroma, or tumor microenvironment (TME), composed of a heterogeneous group of cells including fibroblasts, vascular cells, immune and inflammatory cells. The cancer cells recruit stromal cells from the neighboring normal tissue and activate them into dynamic tumor promoting cells, like myofibroblasts and polarized macrophages (1–3) that contribute to the local cancer tissue morphogenesis. As tumors grow, fibrotic tissue develops and locally enter a hypoxic stage caused by limited access to nutrients and oxygen leading to apoptosis and cell death (4). The tumor-related stroma and extracellular matrix (ECM) reduce drug penetrance and efficacy (5, 6). To understand molecular mechanisms in cancer cells, e.g. linked to drug treatment, cancer cell lines have traditionally been grown in culture flasks in 2D, however, it is evident that such cancer cell lines only poorly recapitulate cancer cells *in vivo*, leading to failure when testing drug candidates in cancer patients (7). Driven by a growing understanding of the need for increased complexity in cellular drug testing models along with the goal of improving the success rate in drug selection programs and minimizing the translational gap between experimental animals and humans, there is a current focus on the development of more complex multicellular tumor spheroids (MCTS). Early three-dimensional (3D) models based on cancer cell monocultures better simulated the *in vivo* situation with respect to physiological barriers caused by intercellular junctions and development of a hypoxic environment (8). Accordingly, IC50 values for chemotherapeutic drugs like 5FU and cisplatin tested in 3D cultures are higher than in 2D cultures (9).

Heterologous 3D *in vitro* cell spheroids composed of breast cancer cell lines and fibroblast cell lines were first reported by Kunz-Schughart and coworkers in 2001 (10) and such co-cultures and their potential as tools in cancer research were later reviewed by Jong Bin Kim (7). During the last decade, several studies have focused on developing more complex cancer spheroids, where different components of the TME are included. Thus, fibroblasts, endothelial and immune cells have been introduced to the cancer cells grown in 3D structures (11). In particular, cancer cell lines grown in co-culture with fibroblasts as 3D hetero-cellular spheroids (heterospheroids) represent interesting novel models for drug testing (11–13). A variety of heterospheroids, a subgroup of MCTS (13), have been developed and comprise human cancer cell lines originating from e.g. colon (14, 15), breast (16, 17), pancreas (18–21), skin (22) and lung (23–26), combined with fibroblast cell lines often derived from human lung (MRC-5 cells) or from mouse embryo (NIH/3T3 cells).

The cultured cancer cells mixed with fibroblasts spontaneously cooperate to form 3D structures when grown in attachment-free round bottom wells (16, 18, 19, 21, 22) or in hanging droplets (23–26). MRC-5 fibroblasts in heterospheroid cultures with lung cancer cells have been shown to introduce ECM components like collagen and laminin in the spheroids (25), thereby, forming a more tumor-like TME. The presence of fibroblasts in the 3D cancer spheroids may also impact on drug efficacy (14).

With the aim of developing 3D cancer models comprising cancer cell and fibroblast cell lines, we here report major structural variety in the spheroid architectural organization linked to the individual cancer cell lines. We show that cancer cell lines confer architectural organization of the fibroblasts within the spheroids, and that both the cancer cells and the fibroblasts contribute to the characteristic spheroid architecture. Molecular analyses indicate that the spheroid co-cultures retain expression of ECM related genes, that cancer related pathways are stimulated and that drug testing using classical chemotherapeutic compounds can be assessed by viability and apoptosis assays. Together, the 3D heterospheroids provide tumor-like structures and the possibility of dissecting drug targeting at the molecular level during pre-clinical drug testing.

## 2 Material and methods

### 2.1 Cell lines

In this study we employed cancer cell lines MCF-7 (ATCC®, HTB-22™), SW480 (ATCC®, CCL-228 ™), HT-29 (ATCC®, HTB-38™), PANC-1 (ATCC®, CRL-1469™), and fibroblast cell lines 1BR.3.G (ECACC, Cat. 90020507), MRC-5 (ATCC®, CCL-171™) and GM00498 (Coriell Institute). Cultures were maintained in Dulbecco’s Modified Eagle’s Medium (DMEM) supplemented with GlutaMAX (Gibco, #32430100) containing 10% fetal bovine serum (FBS) (Biological Industries, Kibbutz Beit-Haemek, Israel, #04–007-1A), 1% Pen/Strep (Lonza, #DE17–602F) and 1% sodium pyruvate (100 mM) (Gibco™, #11360070). Cells were maintained for maximum 20 passages at 37°C in 5% CO_2_ and split weekly or when approaching 90% confluence. The cell lines used in this study are summarized in **Table 1**.

**Table 1:** Cell lines.

#	Cancer cell Line	Sex origin*	Vendor	Cancer type
1	HT-29	F	ATCC	Colorectal Cancer
2	SW480	M	ATCC	Colorectal Cancer
3	HCT116	M	ATCC	Colorectal Cancer
4	MCF-7	F	ATCC	Breast Cancer
5	MDA-MB-231	F	ATCC	Breast Cancer
6	PANC-1	M	ATCC	Pancreatic Cancer
7	MRC-5	M	ATCC	From human Lung
8	1BR.3.G	M	Merck	From human Skin
9	HIF	NA	ScienceCell	From human Intestine
10	GM00498	M	Coriell Institute	From human Skin

*F and M indicate female and male origin, respectively. NA indicate that sex origin of the HIF cells was not available from the vendor.

### 2.2 Spheroid formation

Cells were detached with TrypLE (Gibco, #12605010). For mono-cultures, 2500 cancer cells were seeded per well in ultra-low-attachment (ULA) 96-well plates (Corning®, Corning, NY, USA, #7007) or white PrimeSurface 96U plates (S-bio, #MS-9096WZ) for subsequent viability and apoptosis assays. For co-cultures, 2500 cancer cells and 5000 fibroblasts cells (1:2 ratio) were seeded per well. Cells were centrifuged for 10 min at 500 G and cultured in 200 µL culturing media as described above at 37°C in 5% CO_2_ until spheroid formation and downstream analysis. Media was changed every 3–4 days.

### 2.3 Immunohistochemistry

Spheroids were embedded in paraffin as described previously (27). Five replicate spheroids were placed in each paraffin block and it was attempted to place spheroids uniformly at the same level. Four µm thick tissue sections were obtained and placed on Superfrost Plus glass slides and air-dried overnight. Immunoperoxidase staining was performed using a Ventana Discovery Ultra instrument (Roche, Basel, Switzerland). Rabbit (Rb) anti-smooth muscle α-actin (α-SMA) diluted 1:2,000 (RabMab ab124964), Rb anti-Ki67 diluted 1:12,000 (RabMab ab92742) and Rb anti-collagen-1 diluted 1:3,000 (RabMab EPR7785) were all obtained from AbCam, Cambridge, UK. Mouse (Ms) anti-Mannose Receptor C Type 2 (MRC2, CD280, mAb OTI9G4), diluted 1:10,000 was obtained from OriGene, Rockville MD, USA, and Ms anti-pan cytokeratin (CK, mAb MNF116, M0821) diluted 1:1,000 was obtained from Dako-Agilent, Glostrup, Denmark. The primary antibodies were detected using horse-radish-peroxidase (HRP) conjugated secondary antibodies, OmniMap anti-Ms HRP and OmniMap anti-Rb HRP (Cat. 760–4310 and 760–4311, Roche). Diaminobenzidine (DAB, Roche #760–500) was used as substrate for HRP leading to brown-colored precipitation. Stained slides were scanned with a Zeiss AxioScan bright field scanner (Zeiss, Oberkochen, Germany) using a 20x objective. Representative examples were acquired as TIF images using Zeiss Zen software (RRID: SCR_013672) and processed in CorelDRAW (RRID: SCR_014235).

### 2.4 Immunofluorescence

Automated 4-plex immunofluorescence staining was performed on 4 µm thick paraffin sections from various paraffin embedded cancer spheroids. The strategy for the 4-plex assay was based on the use of Tyramine Signal Amplification (TSA), where the fluorophore substrates are cross-linked locally in the tissue. Consecutive detection of Carbonic anhydrase 9 CA9 (RabMab EPR23055–5 at 1:10,000, AbCam), cleaved Caspase-3 (Rb #9661 at 1:500, Cell Signaling Technologies, Danvers MA), MRC2 (mAb OTI9G4 at 1:10,000, OriGene) and CK (mAb MNF116 at 1:1,000, Dako-Agilent) was performed with OmniMap anti-Ms HRP and OmniMap anti-Rb HRP and the fluorophore TSA substrates DCC, Cy5, Rhodamine and FAM (Roche, #760–240, #760–238, #760–244, #760–243, respectively). In between antibody incubations, primary and secondary antibodies were eluted from sections by incubation in CC2 reagent (Roche, #950–223) at 100°C min for 8 min, before adding the next primary antibody. The stained slides were mounted with DuraTect DAPI-containing mounting medium (ZytoVision Cat #MT-0008–0.8, Bremerhaven, Germany). The stained slides were scanned at 20x using a Pannoramic confocal slide scanner (3DHISTECH Ltd., Budapest, Hungary) equipped with LED light sources and filter sets for DAPI (DAPI), DCC (Aqua), Cy5 (Cy5), Rhodamine (TRITC) and FAM (FITC), see (28). Representative examples were acquired as TIF images using Pannoramic Viewer (RRID: SCR_014424) and processed in CorelDRAW (RRID: SCR_014235).

### 2.5 *In situ* hybridization

mRNA *in situ* hybridization (ISH) was performed using RNAscope probe- and detection technology (29) using a Ventana Discovery Ultra instrument (Roche). RNAscope probes (ACD/Biotechne, Newark, CA) for CLDN4 (Claudin-4, target region: 281–1436, 20 zz pairs, #421049), FAP (fibroblast activation protein α, 237–1549, 20 zz pairs, #411979), PPIB (Cyclophilin B, 139–989, 16 zz pairs, # 313909) and dapB (a Bacillus subtilis gene, 414–862, 10 zz pairs, #312039) were obtained, of which the probes to PPIB and dapB are positive and negative control probes, respectively. The RNAscope probes were detected using the RNAscope™ VS Universal AP (Red) kit (ACD/BioTechne, #323250). Stained slides were scanned with a Zeiss AxioScan bright field scanner (Zeiss, Oberkochen, Germany) using a 20x objective.

### 2.6 RNA-extraction

Total-RNA was extracted from 3D spheroids derived from mono-cultures of SW480, HT-29, PANC-1 and 1BR.3.G after 2 (SW480 and 1BR.3.G), 4 and 7 days of culture and of 3D heterospheroids comprising co-cultures of SW480/1BR.3.G, HT-29/1BR.3.G and PANC-1/1BR.3.G after 2 (only SW480/1BR.3.G), 4 and 7 days of culture. For each condition, RNA was extracted using the Single Cell RNA Purification Kit (Cat. #51800, Norgen Biotek Corporation, Thorold, ON, Canada). This kit is designed for efficient extraction of RNA from cell cultures with less than 2 x 105 cells. The spheroids were carefully transferred to an RNase-free Eppendorf tube. Centrifugation at 5000 g for 5 min was performed and excess medium was removed before adding the cell lysate buffer following the manufacturer’s instruction from here on. Following extraction, the concentration of the RNA was determined using the Quant-iTTM RiboGreenTM RNA Reagent and Kit (Cat. #R11490, ThermoFisher Scientific, Roskilde, Denmark) according to the manufacturer’s instructions.

### 2.7 Whole transcriptomic analysis, RNA sequencing

RNA-samples representing each condition were sent to an external service provider (Novogene, Cambridge, UK) performing human mRNA sequencing using the Illumina platform NovaSeq PE150 with paired-end 150 bp sequencing strategy. All samples passed the quality control system of the service provider, and samples and RNA-seq was performed on equal amounts of all samples generating 6 GB raw data per sample.

### 2.8 Bioinformatic analyses and graphics of WTA data

Standard data analysis was performed by the service provider including, data quality control and data filtering, mapping to reference genome (Homo_sapiens_Ensemble_94), gene expression quantification and enrichment analysis (GO and KEGG). The full data package was delivered and gene expression data for selected genes across all samples were extracted from the data set. For graphical presentation of WTA data we used OriginPro (RRID: SCR_014212, Northampton, MA).

### 2.9 Drug exposure

Drug exposure was performed as described by Selby et al. (30). Prior to drug intervention, cells were seeded as described above and allowed to incubate for 72 hr in order for spheroids to form. To examine the response to anticancer drugs cisplatin (Merck #C2210000), and 5-fluorouracil (5-FU) (Sigma-Aldrich #F6627), dilution series of the drugs were prepared from dimethylformamide (DMF) stock solutions of cisplatin (33.3 mM) and 5-FU (461 mM) in culture media. Dilutions were made to allow for the addition of 6 µl drug suspensions to 190 µl of medium with the spheroids to reach the indicated final concentrations. Vehicle suspension contained 3.54% (v/v) DMF in culture medium. Drug exposure was performed for 72 hr before assessment of drug effects using both a viability and an apoptosis assay. The HT-29 heterospheroid model was used for testing the NCI drug library version AOD X, kindly provided by the National Cancer Institute Developmental Therapeutic Program (NCI/DTP). This library consists of 166 approved oncology drugs, and the drugs were added at a final concentration of 10 µM on day 4 after seeding. After 72 hr of drug exposure, drug effects were evaluated by bright-field imaging, viability and apoptosis assays as previously described.

### 2.10 Viability assay

After drug exposure the viability of the spheroid cells was assessed using the CellTiter-Glo® 3D Cell Viability Assay (Promega #G9681) following the manufacturer’s instructions. In brief, one hundred µl of the medium in the spheroid wells were carefully removed without affecting the spheroids, and subsequently a volume of the CellTiter- Glo® reagent equal to the remaining cell culture medium (100 µl) in the wells was added. The plate was sealed and mixed vigorously for 5 min and then allowed to incubate for another 25 min. before reading the luminescent signal in a Tecan Infinite 200Pro luminometer (RRID: SCR_020543). Drug response curves were prepared in GraphPad Prism (RRID: SCR_002798) using a variable slope model based on a non-linear fit least square using 4 variables.

### 2.11 Apoptosis assay

After drug exposure caspase 3/7-activation was evaluated as a measure of drug toxicity. The assay buffer was prepared comprising 150 mM HEPES buffer, pH 7.4, 450 mM NaCl, 150 mM KCl, 30 mM MgCl_2_, 1.2 mM EGTA, 1.5% (v/v) Nonidet P40, 0.3%(w/v) CHAPS, 30%(w/v) sucrose. Just before use, 90 µL 10 mM N-acetyl-DEVD-7-amino-4-methylcoumarin (DEVD-AMC) peptide (final 150 µM), 180 µL 1M DTT (final 30 mM) and 30 µL 200 mM PMSF (final 1,0 mM) was added to 5.70 mL assay buffer (6 mL total volume hereafter). One hundred µl of the medium in the spheroid wells were carefully removed without affecting the spheroids, and 50 µL of assay buffer was added to each well and incubated at 37°C for 1 hr in the dark. The caspase-3/7-activity (31) was measured by reading proteolytically released fluorochrome from the DEVD-AMC substrate using a Tecan Infinite 200Pro fluorometer (RRID: SCR_020543) with an excitation at 360 nm and an emission at 460 nm.

### 2.12 Statistics

Comparing metabolic and signal transduction pathways in the WTA data from HT29/1BR.3.G heterospheroids and HT-29 spheroids was done by conventional KEGG (Kyoto Encyclopedia of Genes and Genomes) enrichment analysis. The 20 most significantly enriched KEGG pathways (P<0.05) are depicted in **Figure 4D**. Drug exposure assays using cisplatin and 5-FU were all performed in triplicate and when fitting drug response curves (**Figure 6A**), the fluorescence and luminescence intensities associated with caspase 3/7 activation and CellTiter-Glo® 3D viability assay, respectively, were depicted as the mean of triplicate measurements +/- standard deviation. For drug testing in biological replicates, unpaired T-test was applied to assess the difference between treatment groups (assuming a Gaussian distribution of data points), **Supplementary Figure S3B**. The tests and graphics were done using GraphPad Prism.

**Figure 1:**
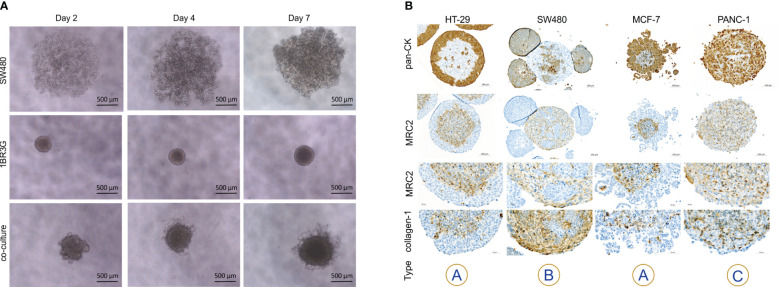
Architectural organization of cancer cell heterospheroids. **(A)** Brightfield images of SW480 cancer cells and 1BR.3.G fibroblasts grown separately and as co-culture at days 2, 4 and 7 after seeding in ULA plates. Note that the cells are assembled into dense spheroids when co-cultured. **(B)** Immunoperoxidase staining for the cancer cell marker cytokeratin, the fibroblast marker MRC2 and collagen type-1 on paraffin embedded spheroids. HT-29 and MCF-7 cells grow in the periphery of spheroids with 1BR.3.G cells in the core (type A). SW480 cells are surrounded by the 1BR.3.G fibroblast in (type B) if not growing as satellites on top of the spheroid. PANC-1 cells intersperse with the 1BR.3.G fibroblasts to form type C heterospheroids. Abundant collagen staining is associated with the fibroblasts. The examples are representative of 4–5 replicate spheroids in each paraffin block.

**Figure 2:**
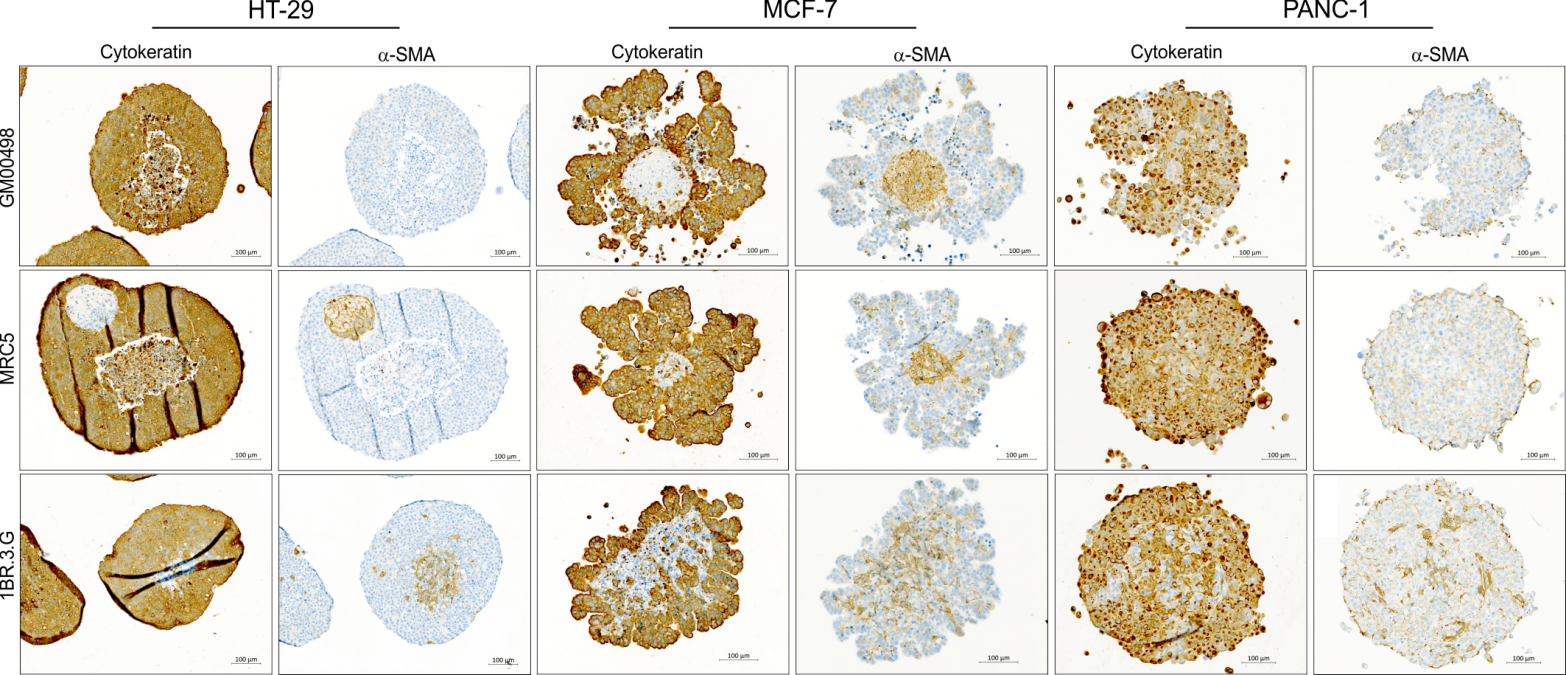
Heterospheroids formed by fibroblast cell lines. Heterospheroids were obtained from HT-29, MCF-7 and PANC-1 cells co-cultured with GM00498, MRC-5 or 1BR.3.G fibroblasts. The spheroids were embedded in paraffin and sections stained for pan-cytokeratin and the fibroblast marker α-SMA. GM00498 and MRC-5 fibroblasts form a central core (α-SMA positive) when growing with MCF-7, but are not cooperating with HT-29 and PANC-1 cells. MRC-5 fibroblasts form a separate structure (α-SMA positive) when grown together with HT-29, and the HT-29 spheroid show a necrotic core. 1BR.3.G fibroblasts form a central core when growing with HT-29 and MCF-7 (α-SMA positive), and intersperse with the PANC-1 cells. The examples are representative of 4–5 replicate spheroids in each paraffin block.

## 3 Results

### 3.1 Cancer cell lines utilize fibroblasts to shape the architecture of 3D spheroids

**Figure 3:**
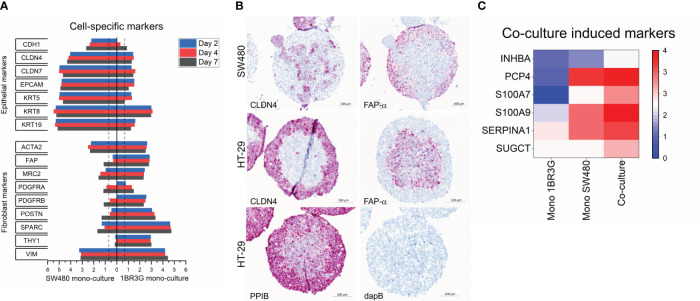
Molecular characteristics of SW480 spheroids. Total RNA was obtained from spheroids of SW480, 1BR.3.G and their co-cultures at days 2, 4 and 7 after seeding in ULA plates, and submitted to WTA. **(A)** Expression of typical epithelial and fibroblast markers in the SW480 3D monocultures (left) and 1BR.3.G 3D monocultures (right). Note that ACTA2 (α-SMA) and VIM (vimentin) are relative high in SW480 monocultures suggesting some level of EMT in the SW480 cells. Expression levels are log_10_(100*FPKM) transformed values. Only values above 0.05 FPKM (ca. 0.07) are considered significant (dotted vertical lines). **(B)** *In situ* hybridization for the epithelial marker Claudin 4 (CLDN4) and the fibroblastic marker FAP-α (red stain) in the SW480 heterospheroid and an HT-29 heterospheroid as reference. Positive control probe (PPIB) and negative control probe (dapB) are shown in the case of HT-29 heterospheroids. Hematoxylin counter staining. **(C)** Heat map showing the upregulated expression of genes in SW480/1BR.3.G co-cultures (Co) compared to the 1BR.3.G and SW480 3D monocultures (red indicates relatively higher expression per cell).

The cell lines investigated are listed in **Table 1**, which also indicates the sex of the donors of the cell lines and the tissue of origin. We have previously reported that 1BR.3.G skin fibroblasts (32) facilitate spheroid formation with a variety of human cancer cell lines, including HT-29, MCF-7, and PANC-1 (33, 34). In this work, the KRAS^G12V^ mutant colon cancer cell line SW480 was also added to the panel, as we found that 1BR.3.G fibroblasts clearly enhanced spheroid formation with this cell line (**Figure 1A**). The mechanism of how fibroblasts support spheroid formation is not known, but the fibroblasts likely produce an ECM that the cancer cells adhere to, and use their actin filaments to contract the cell populations. To characterize the morphological structures of the heterospheroids with these four cell lines we performed immunohistochemical staining of pan-cytokeratin and MRC2/CD280 (35) to stain cancer cells and fibroblasts, respectively. Staining of MRC2 was found to be more fibroblast-specific than staining of α-SMA (in accordance with mRNA expression data, see **Figure 3**), however, in most cases we stained for both MRC2 and α-SMA. The four cancer cell lines formed three distinct types of architectural structures when cultured with the 1BR.3.G fibroblasts. The HT-29 and MCF-7 cells surrounded a fibroblast-rich spheroid core (structure type A), whereas the SW480 cells formed a core surrounded by the fibroblasts (structure type B) (**Figure 1B**). SW480 cells also formed peri-spheroid appendices of propagating cells. The PANC-1 cells interspersed with the fibroblasts with no obvious organization (structure type C). These observations suggest that the cancer cell lines communicate with fibroblasts in different ways to define and shape the architectural organization of the spheroids. The fibroblast population to a varying degree also expressed collagen-1 in the heterospheroids (**Figure 1B**).

**Figure 4:**
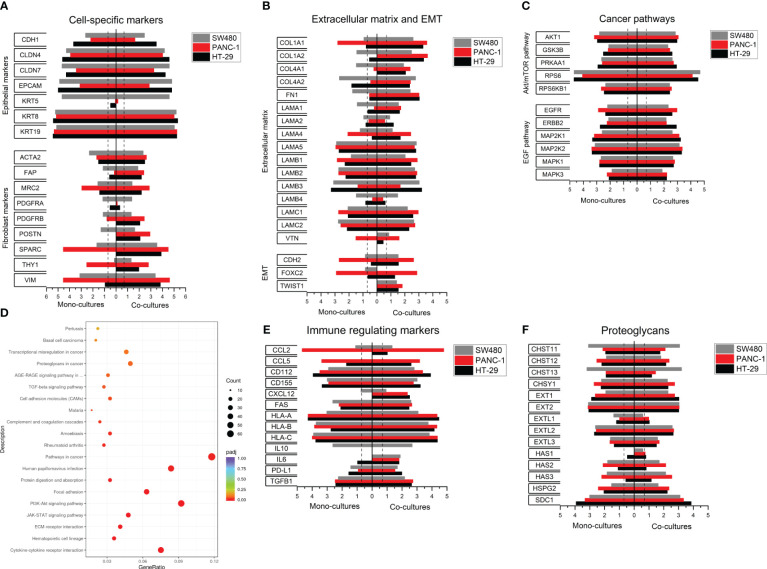
Expression profiling in SW480, HT-29 and PANC-1 heterospheroids. Total RNA was obtained from spheroids of SW480, PANC-1 and HT-29 and their co-cultures with 1BR.3.G at day 7 after seeding in ULA plates, and the RNA submitted to WTA. **(A)** Selected expressed genes of epithelial and fibroblast markers in 3D cancer cell monocultures (left) and 3D heterospheroids with 1BR.3.G fibroblasts (right). **(B)** Selected expressed genes related to ECM and EMT. **(C)** Selected expressed genes related to cancer pathways. **(D)** Gene function (KEGG) analysis of WTA from HT-29/1BR.3.G vs. HT-29 spheroids. The abscissa represents the ratio of the number of differentially expressed genes linked with the KEGG pathway to the total number of differentially expressed genes. The ordinate represents the 20 most significant KEGG Pathways. The size of a point represents the number of genes annotated to a specific KEGG pathway. The color from red to purple represents the significance level of the enrichment. **(E)** Selected expressed genes related to immune modulation. **(F)** Selected genes related to glycosylation. Expression levels are log_10_(100*FPKM) transformed values. Only values above 0.05 FPKM (ca. 0.07) are considered significant (dotted vertical lines).

### 3.2 The fibroblast cell line is key to support spheroid formation

The 1BR.3.G fibroblasts were found easy to handle and they proliferated well in standard growth medium (DMEM) that was compatible with the cancer cell lines used. To test if other fibroblast cell lines promoted similar architectural organization as the 1BR.3.G cells, each of six cancer cell lines (HT-29, SW480, MCF-7, PANC-1, MDA-MB-231, and HCT-116) were co-cultured with GM00498 (skin fibroblast) or MRC-5 (lung fibroblast) cell lines in ULA plates for 12 days in parallel with 1BR.3.G fibroblasts. MRC-5 and GM00498 fibroblasts, as identified by α-SMA immunohistochemistry, supported the formation of spheroid structures with the MCF-7 cells (**Figure 2**). The MRC-5 cells were also identified in co-cultures with HT-29, PANC-1, MDA-MB-231 and HCT-116, but they were few in numbers compared to spheroids incorporating 1BR.3.G fibroblasts (**Figure 2** and **Supplementary Figure S1**). In HT-29 spheroids, the MRC-5 fibroblasts formed an isolated structure surrounded by the HT-29 cells (**Figure 2**). The GM00498 fibroblasts were found in spheroids formed by MCF-7 and HCT-116, but were all lost during the co-culture with HT-29, PANC-1 and MDA-MB-231 (**Figure 2** and **Supplementary Figure S1**). The lower prevalence of α-SMA positive fibroblasts suggests that the growth rate of the MRC-5 and the GM00498 fibroblasts was suppressed in the spheroid co-cultures. In addition, the human intestinal fibroblasts (HIF) cell line was tested in co-cultures with either HT-29 or SW480 cancer cells, but the HIF cells poorly supported spheroid development. Like the MRC-5 fibroblasts, the HIF cells formed isolated structures of fibroblasts surrounded by HT-29 cells, and the HIF cells were not present after 12 days together with SW480 cells (data not shown). Thus, although both MRC-5 and GM00498 fibroblasts supported spheroid formation with some of the cancer cells, the 1BR.3.G cells were those that supported the heterospheroid growth the best and generated *in vivo*-like morphology representing tumor tissue.

**Figure 5:**
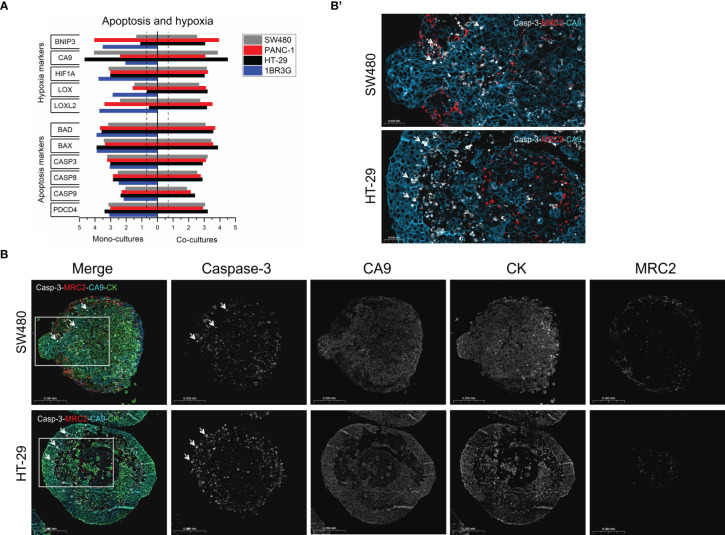
Apoptosis and hypoxia in HT-29 and SW480 heterospheroids. **(A)** Total RNA was obtained from spheroids of SW480, PANC-1 and HT-29 and their co-cultures with 1BR.3.G at day 7 after seeding in ULA plates, and submitted to WTA. Selected expressed genes related to hypoxia and apoptosis are shown. Expression levels are log_10_(100*FPKM) transformed values. Only values above 0.05 FPKM (ca. 0.07) are considered significant (dotted vertical lines). **(B)** Paraffin sections from SW480 and HT-29 were stained in a 4-plex immunofluorescence assay to cleaved Caspase-3 and CA9 together with cytokeratin and MRC2 to show the cancer and fibroblasts, respectively. Whereas CA9 is well expressed in the majority of cancer cells, cleaved Caspase-3 is upregulated in a subset of cells. In SW480 heterospheroids, cleaved Caspase-3 is seen scattered within the structure and primarily in the cancer cells (examples indicated by arrows), and in HT-29 cleaved Caspase-3 is seen most intensely in cancer cells located towards necrotic core region (examples indicated by arrows). **(B’)**. The framed area in B (Merge) shows cleaved Caspase-3, CA9 and MRC2.

### 3.3 Characterization of SW480 heterospheroids

Because of the particular structure of SW480 heterospheroids, we went on to characterize the expression dynamics of SW480 and 1BR.3.G spheroid mono- and co-cultures using WTA on RNA isolated on day 2, 4, and 7 after seeding. The repeated measures (on days 2, 4 and 7) indicated consistent expression levels of epithelial and fibroblastic markers in the SW480 and 1BR.3.G cell mono-cultures, respectively (**Figure 3A**). The transcriptomic analysis confirmed mRNA expression of classical epithelial markers (cytokeratins, claudins, EpCAM, E-cadherin) in the SW480 cancer cell mono-spheroids and typical fibroblast markers (ACTA2/α-SMA, FAP-α, PDGFR, and MRC2) in the fibroblast mono-spheroids. The expression levels of these epithelial and fibroblast markers (**Figure 3A**, right) were retained in the co-cultures during spheroid growth (see **Figure 4**). To validate the cell specific expression, we performed RNA *in situ* hybridization for Claudin-4 and FAP-α, which were, as expected, seen in the SW480 cancer cells and the 1BR.3.G fibroblasts (**Figure 3B**), respectively. Interestingly, the transcriptomic analysis showed that the spheroid co-culturing, compared to separate mono-cultures of HT-29 and fibroblasts, induced upregulation of INHBA, SerpinA1 and S100A9 (**Figure 3C**), which are well-known players in tumor biology (36–38).

### 3.4 ECM and EMT are characteristics of heterospheroids

The HT-29 and PANC-1 heterospheroids that formed the architectural organization type A and C, respectively, were then submitted to WTA. Selected data from 7-day-old HT-29 and PANC-1 spheroid mono- and co-cultures, as well as the data from the day-7 SW480 spheroid mono- and co-cultures (**Figure 3**), are assembled in **Figure 4**. Evaluating epithelial and fibroblast markers (**Figure 4A**), the HT-29 spheroid co-cultures showed a similar expression profile to SW480. Among the epithelial markers listed, SW480 had a high level of KRT5 and a low level of E-cadherin (CDH1) as opposed to HT-29 spheroids. In contrast, PANC-1 spheroid co-cultures exhibited lower levels of epithelial markers and higher levels of fibroblast markers, suggesting that the PANC-1 cells have a more mesenchymal phenotype. This is supported by the increased levels of the fibroblast markers, such as SPARC and Vimentin (**Figure 4A**), and increased levels of the epithelial-mesenchymal-transition (EMT) markers FOXC2 and CDH2 (**Figure 4B**).

Among the listed ECM genes (**Figure 4B**), two types were noted, those that were low in mono-cultures and high in co-cultures (e.g. COL1A, FN, LAMA1, primarily fibroblast-derived) and those that were high in both mono- and co-cultures (e.g. LAMA5 and LAMB1, primarily cancer cell-derived). The abundant ECM expression in the co-cultures reflects the presence of fibroblast, and thus indicates the more tumor-like molecular profile. In addition, the three co-cultures expressed abundant levels of genes related to key cancer pathways, EGF and AKT/mTOR (**Figure 4C**), with no obvious difference between the three cancer cell lines. KEGG enrichment and pathway analysis of the expression data also indicated the abundant expression of genes related to cancer pathways when comparing HT-29 heterospheroids with HT-29 mono-cultures (**Figure 4D**) emphasizing the high biological activity of these pathways.

We also analyzed a list of genes related to interactions with immune cells (**Figure 4E**), as heterospheroids can be submitted to immune cell infiltration studies (34). We noted increased levels of PD-L1 expression in all three heterospheroids, SW480, PANC-1 and HT-29, which potentially would affect T cell function (39). In addition, obvious differences in the expression of CCL2, CCL5 and CXCL12, that are associated with the recruitment of immunosuppressive cells (40–42), were noted among the three heterospheroids, and this information would be helpful for selecting a proper model for immuno-oncology studies. Finally, we found major differences among the cancer cell lines in their expression of genes involved in glycosylation, including CHST11, CHST13, Ext1, HAS2 and HAS3, which may have an impact on the architectural organization of the spheroids (**Figure 4F**).

### 3.5 Hypoxia-related gene expression and cell death in HT-29 and SW480

Hypoxia is a well-known characteristic of cancer spheroids when these exceed just a few hundred micrometers (43). A key feature that is shared with patient tumors. Expression levels of a list of selected hypoxia and apoptosis-related genes are shown in **Figure 5A**. The 1BR.3.G fibroblasts and the three cancer cell lines expressed several hypoxia-related genes. However, CA9 was dominating in the cancer cell lines, whereas LOX and LOXL2 were dominating in the fibroblasts. The apoptosis-related genes were more evenly expressed among the cell lines. To better understand presence of apoptosis and hypoxia, we stained the apoptosis-induced cleaved Caspase-3 (active Caspase-3) and the hypoxia-marker CA9 together with cytokeratin and the fibroblast marker MRC2 in 12-day-old heterospheroids of HT-29 and SW480 cancer cells using 4-plex immunofluorescence (**Figure 5B**). In both heterospheroids, CA9 immunofluorescence staining was primarily associated with the cancer cells, but also some of the fibroblasts were CA9 positive. Immunofluorescence staining of cleaved Caspase-3 was seen in the necrotic core area of HT-29 heterospheroids with the most intense staining in individual cancer cells located at the edge of necrotic areas. In SW480 heterospheroids, cleaved Caspase-3-positive cells were more evenly distributed throughout the spheroids and was seen in both cancer cells and fibroblasts. Thus, these results not only confirmed that hypoxia is a major characteristic of the heterospheroids, but also that the hypoxic stage did not cause excess apoptosis.

**Figure 6:**
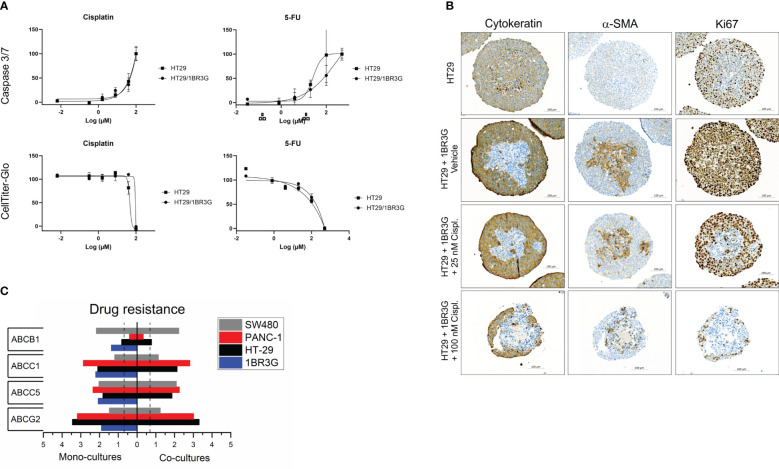
Cisplatin and 5FU treatment of heterospheroids. **(A)** Cisplatin or 5FU were added to day-4 HT-29 spheroids with and without 1BR.3.G fibroblast. Three days after, the toxicity was measured by a Caspase 3/7 activity assay and a cell viability assay (CellTiter-Glo 3D). **(B)** Immunohistochemical staining of pan-CK, α-SMA and Ki67 in paraffin sections from HT-29 spheroids with and without 1BR.3.G fibroblasts. Spheroids were treated with vehicle, low and high dose cisplatin. **(C)** WTA data on selected drug resistance genes. Total RNA was obtained from spheroids of day-7 mono- and co-cultures as indicated. Expression levels are log_10_(100*FPKM) transformed values. Only values above 0.05 FPKM (ca. 0.07) are considered significant (dotted vertical lines).

### 3.6 Drug testing in heterospheroids

Cisplatin and 5FU are common cytotoxic therapeutic agents that target proliferating cells. The effects of these drugs on 2D vs 3D cell cultures have previously been analyzed using cell viability assays (9). Here, we used both a cell viability and an apoptosis assay to obtain two independent parameters of the cytotoxic agents. In order to test the effect of the presence of 1BR.3.G fibroblasts in spheroids, we selected the HT-29 model since this also form spheroids without the presence of fibroblasts. Testing the two drugs in HT-29 mono-cultures and HT-29 co-cultures (**Figure 6A**), we found no major differences in the cytotoxicity. In parallel with the spheroids used in the cell viability and the apoptosis assays, we obtained representative spheroids for immunohistochemical analysis, thus HT-29 mono-spheroids and HT-29/1BR.3.G heterospheroids as untreated or treated with low (25 µM) and high (100 µM) dose of cisplatin. Consecutive sections were then stained for CK, α-SMA and Ki67 (**Figure 6B**). Both the HT-29 cancer cells and the 1BR.3.G fibroblasts expressed the Ki67 proliferation marker (**Figure 6B**, right column), making both cell populations target for chemotherapeutic compounds. The immunohistochemical analysis indicated that the fibroblasts were targeted at low-dose-cisplatin since their nuclei became fragmented and they lost the Ki67 staining (**Figure 6B**, row 3), whereas no obvious effect was seen in the cancer cells. At high-dose-cisplatin (100 µM), both the HT-29 cancer cells and fibroblasts were killed by the drug and the remaining spheroid structures were small and ruptured (**Figure 6B**, row 4). Our expression data indicated that HT-29 cells expressed low levels of ABCB1 (MDR1) and high levels of the ABC transporter ABCG2, whereas the 1BR.3.G fibroblasts expressed relatively low levels of both resistance genes (**Figure 6C**), which may suggest that drug resistance mechanisms can explain why the fibroblasts are more sensitive to the drug.

To test the reproducibility and similarity of the drug response caused by cisplatin in the HT-29 heterospheroid model, we compared three different treatments; vehicle, 25 µM and 100 µM cisplatin in groups of twenty HT-29 heterospheroids and recorded their macroscopic structure before (at T=0) and after drug exposure (T=72 hrs). The cisplatin-treated spheroids strongly changed morphologic characteristics, in particular by losing structural integrity in the periphery (**Supplementary Figure S3A**), which probably was caused by dying cells detaching from the main structure. The spheroids treated with vehicle appeared unaffected and increased in size during the 72 hrs. Indeed, the readout measurements of the Caspase 3/7 assay showed dose-dependent increased activity after 72 hrs (**Supplementary Figure S3B**).

We then tested the versatility of our 3D heterospheroid models, by exploring the effect of the 166 approved oncology drugs from the AOD X plate set (provided by NCI/DTP) using HT29 heterospheroids as model. Bright-field images were acquired after 72 hrs followed by viability and apoptosis assays. Increased apoptosis (defined as more than the average response of vehicle plus three times the standard deviation) was measured in 103 of the 166 drugs (62%), and reduced viability (defined as less than the average response of vehicle minus three times the standard deviation) was measured in 73 of the drugs (44%). Examples from 5 different drugs (Doxorubicin, Bortezomib, Belinostat, Crizotinib and Cobimetinib) are shown in **Supplementary Figure S4**. The drugs strongly affected the integrity of the spheroids (**Supplementary Figure S4A**), and variably induced cell death (apoptosis) (**Supplementary Figure S4B**) and reduced viability (**Supplementary Figure S4C**).

## 4 Discussion

With the aim of developing human 3D cancer models composed of the two key cell components in most adenocarcinomas, cancer cells and fibroblasts, we here report major architectural variety in the spheroid structure linked to individual cancer cell lines. Surprisingly, we found that different cancer cell lines consistently promoted different architectural organization, thus interspersing, surrounding or being subservient to the fibroblasts, and at the same time retaining expression of ECM-related genes. Overall, we found that co-culture spheroids retain expression of tumor-relevant genes compared to mono-culture spheroids. These varying structures may be advantageous in anti-cancer drug screenings, e.g. when targeting either cancer cell or fibroblast pathways or even ECM components. We could measure cell viability and apoptosis as two independent output parameters after introducing cytotoxic drugs, suggesting that heterospheroid 3D cancer models are useful for pre-clinical drug screening.

The formation of reproducible 3D cell structures comprising cancer cell lines and the 1BR.3.G fibroblasts indicated dynamic interactions between the two cell populations. We and others have previously reported that fibroblasts support the development of spheroid structures (17, 23, 25, 33, 34), but the use of 1BR.3.G in 3D cancer models has not previously been reported. Here, we found that the 1BR.3.G cell line generally supported 3D spheroid structures to a broader extent than the skin fibroblast GM00498, and the more commonly used lung fibroblast MRC-5, as well as the intestinal HIF fibroblasts. The different performance of the fibroblast cell lines could not be explained simply by sex origin relations or origin of organ, suggesting that some molecular characteristics of 1BR.3.G facilitates heterospheroid formation. Indeed, the different ways that our fibroblast cell lines interacted with cancer cell lines may be crucial for studies of cell-cell interactions. In addition to the origin of the fibroblasts, the method of immortalization and passage numbers, may be key factors for the intrinsic characteristics of the fibroblasts. Further characterization of our fibroblast lines will be needed to identify central molecular players in the inter-cellular dynamics. Fibroblasts may be cultured in a specialized growth medium different from the cancer cell lines, suggesting that DMEM, which was used as standard growth medium during spheroid formation, may differently impact on the fibroblasts’ ability to support 3D spheroid growth. The culture conditions supporting heterospheroid growth are based on multiple variables in addition to the growth medium, such as the cell culture confluence state of pre-cultures, cell mixing, cell-cell ratios, as well as passage numbers. In this study, we aimed at keeping these parameters constant in order to retain a consistent output. However, it cannot be excluded that certain culture conditions may impact on the performance of individual fibroblast cell lines in their ability to support heterospheroid formation.

We found that the 1BR.3.G fibroblast supported SW480 spheroid development that was characterized by the fibroblast surrounding the cancer cells (type B). SW480 cancer cells have also been used in other 3D studies (9, 44), however, not reported elsewhere in combination with fibroblasts. In addition, the ability of fibroblasts to surround cancer cells has to our knowledge not been reported previously. Interestingly, Österholm et al. (23) found that the glycosyltransferase, Ext1, expressed in an embryonic fibroblast cell line, affected the cellular organization of the heterospheroid, probably by interfering with collagen attachment. In that study, the authors found that A549 lung cancer cells were scattered located inside the spheroid if surrounded by mutated fibroblasts with reduced Ext1 expression (Ext1^Gt/Gt^). Our expression data indicated that SW480 heterospheroids express relatively low levels of Ext1 compared to HT-29 and PANC-1, suggesting that protein glycosylation may indeed contribute to architectural organization. Another parameter that may help to explain the different architectural structures is that the SW480 cells express the KRAS^G12V^ mutation, different from the HT-29 cells, which are KRAS^wt^. However, PANC-1 cells are also KRAS mutant (KRAS^G12D^), suggesting that KRAS mutations alone cannot explain the difference in heterospheroid structure. Furthermore, our expression analysis identified KRT5 to be highly expressed in SW480 cells, and absent in HT-29 and PANC-1 cells. KRT5, or cytokeratin 5, is typically expressed in basal cells such as myoepithelial and basal keratinocytes, and is uncommon in colorectal cancer. Only few reports have described adenosquamous tumors of the colon with KRT5 positive expression (45). SW480 may thus be an interesting but atypical colon cancer cell line not representing the majority of colon cancers. It is tempting to speculate that cancer cell lines with squamous characteristics, such as those from skin, lung, cervix and esophagus, would form similar architectural organization in fibroblast-enhanced heterospheroids.

The presence of fibroblasts in the 3D cancer models provides the possibility of testing cancer drugs directed against the fibroblasts themselves, e.g. PDGFR-β and FAP-α that were both expressed in the 1BR.3.G fibroblast, are potential therapeutic targets in cancer (46). The fibroblast-supported spheroids not only facilitated the spheroid formation but also supplied the spheroids with abundant ECM. Here we found expression of a majority of ECM components, including collagens, laminins, and fibronectin. The 3D cancer models thus contain general ECM components typical for the majority of adenocarcinomas. The presence of ECM in the 3D cancer cell models also provides the possibility of testing drug candidates directed against ECM proteins (47). In addition, testing drug penetration through a fibroblast-rich stromal compartment, may be explored in the heterospheroid based models. Indeed, nanoparticle penetration has been studied in heterospheroids (17) as well as the use of drug candidates in combination with a penetration-enhancing compound lowering tumor stiffness (16). Also therapeutic targets directed against EMT-related cell profiles is an option using the cancer heterospheroids. We listed genes that were known for their involvement in EMT, such as CDH2 (N-cadherin), SPARC and Vimentin, and found that the PANC-1 cells expressed several of these genes in contrast to HT-29 and SW480. The EMT characteristics of PANC-1 cells have also been reported by others (48, 49). We believe that the expression of these genes may help to explain the characteristic structure of the PANC-1/1BR.3.G heterospheroids.

Hypoxia is an inherent part of growing cell spheroids and is often linked to increasing central necrosis during growth. Importantly, however, *in vivo* tumors also exhibit hypoxia, and is associated with EMT and poor prognosis (50), making hypoxia an important parameter to consider also in heterospheroids. We noted abundant expression of the hypoxia-related marker CA9 in the HT-29 and SW480 cancer cell monocultures and heterospheroids, whereas the fibroblasts expressed a low level. In contrast, the fibroblasts expressed relatively higher levels of LOX and LOXL2, suggesting that the fibroblasts exhibit a different hypoxia expression profile than the cancer cells. Despite the prevalent expression of hypoxia-related genes, and more restricted expression of cleaved Caspase-3, the heterospheroids retain growth, thus suggesting a robust phenotype of the cell populations. These characteristics of heterospheroids may be helpful for further exploring tumor-associated hypoxia and the impact on EMT.

In order to develop methods to assess the cytotoxic effects, we explored the impact of two well-known drugs, 5FU and cisplatin, and found that both the viability assay and the apoptosis assay measured the cytotoxicity of the drugs. Although the viability assay did not directly measure killing, but the reciprocal events, and that the caspase 3/7 assay only measured apoptosis and not non-apoptosis related cell death, the two assays supplemented each other in the determination of drug effect. The effect of adding fibroblasts to the HT-29 spheroids had minor effect on the cytotoxicity of cisplatin and 5FU, suggesting that the fibroblasts may add complexity to the 3D cancer cell model without affecting the cytotoxicity. In our toxicity analysis of cisplatin, which is a drug that targets dividing cells, in HT-29 heterospheroids, we noted that the fibroblast population appeared more sensitive to the drug than the HT-29 cancer cells, because they lost the expression of the proliferation marker Ki67. Even though the fibroblasts derive from normal tissues, this observation may not seem surprising since these immortalized fibroblasts were highly proliferating in both 2D and 3D monocultures. In addition, the fibroblasts expressed low levels of drug resistance genes, like ABCG2, that could make them appear more sensitive to some drugs than cancer cell lines, as in the analysis of HT-29 heterospheroids (**Figure 6**). In a study of fibroblast-supported Caco-2 spheroids, it was reported that the fibroblast co-cultures decreased resistance to 5-FU/irinotecan, but had no impact on 5-FU/oxaliplatin (14). In this study, we explored the possibility of testing larger panels of drugs, and obtained the NCI/AOD X drug library consisting of 166 approved oncology drugs. Many of the drugs of the NCI/AOD X affected the spheroid integrity, and cell death could indeed be measured for more than 60% of the drugs. The drug screening also indicated a lack of correlation between the viability and cell death assays, suggesting different modes-of-action of different drugs or suboptimal drug concentration. More studies will be needed for a better understanding of these readouts.

Development of useful 3D cancer models for drug testing is currently in the exploratory phase, however, the potential of “three-dimensional tissue culture models” in cancer biology was already discussed in 2005 (7). At least, the fibroblast-enhanced heterospheroids appear as promising tools to mimic *in vivo* tumors. This far, we also know that monocytes from PBMCs become activated and can be found as macrophages within the spheroids (34), resulting in even more complex 3D cancer models. Currently, we also work on adding more immune subsets to our 3D cancer models. Dependent on the mode of action, customized 3D cancer models may be developed for multiple drug types that include small molecules, biologics, antibody-drug-conjugates (ADC) or antisense oligonucleotides (ASO). The heterospheroids may prove particularly useful for cell specific targeting of cancer cells and fibroblasts.

## Data availability statement

The Expression profiling (WTA) data generated in this study are available upon request from the corresponding author.

## Ethics statement

Ethical approval was not required for the studies on humans in accordance with the local legislation and institutional requirements because only commercially available established cell lines were used.

## Author contributions

BN: Conceptualization, Methodology, Project administration, Resources, Supervision, Validation, Visualization, Writing – original draft, Writing – review & editing. NM: Conceptualization, Formal analysis, Investigation, Resources, Validation, Visualization, Writing – review & editing. JL: Conceptualization, Investigation, Methodology, Supervision, Writing – review & editing. IS: Conceptualization, Investigation, Writing – review & editing. MG: Conceptualization, Methodology, Supervision, Writing – review & editing. KH: Conceptualization, Formal analysis, Investigation, Methodology, Resources, Validation, Visualization, Writing – review & editing.
