# Supplementary material for: Architectural organization and molecular profiling of 3D cancer heterospheroids and their application in drug testing
Source: Front Oncol. 2024 Jul 1;14:1386097. doi: 10.3389/fonc.2024.1386097 (PMC11246882; doi:10.3389/fonc.2024.1386097)

Supplementary Material

Architectural organization and molecular profiling of 3D cancer heterospheroids and their application in drug testing

Boye Schnack Nielsen*, Natasha Helleberg Madsen, Jesper Larsen, Isabella Skandorff, Monika Gad, Kim Holmstrøm

*** Correspondence:** Boye Schnack Nielsen, Bioneer, Kogle Allé 2, 2970 Hørsholm, Denmark.

BSN@Bioneer.dk

# Supplementary Figure S1

**Heterospheroids formed by fibroblast cell lines.** Heterospheroids were obtained from HCT‐116 and MDA‐MB‐23 cells co‐cultured with GM00498, MRC‐5 or 1BR.3.G fibroblasts. The spheroids were embedded in paraffin and used for immunohistochemical staining of cytokeratin and the fibroblast marker α‐SMA. MRC‐5 fibroblasts form a central core (α‐SMA positive) when growing with HCT‐116, but are not cooperating with MDA‐MB‐231 cells. The GM00498 fibroblast do not support MDA‐MB‐231 and are suppressed when growing with HCT‐116 cells. The examples are representative of 4‐5 replicate spheroids in each paraffin block.


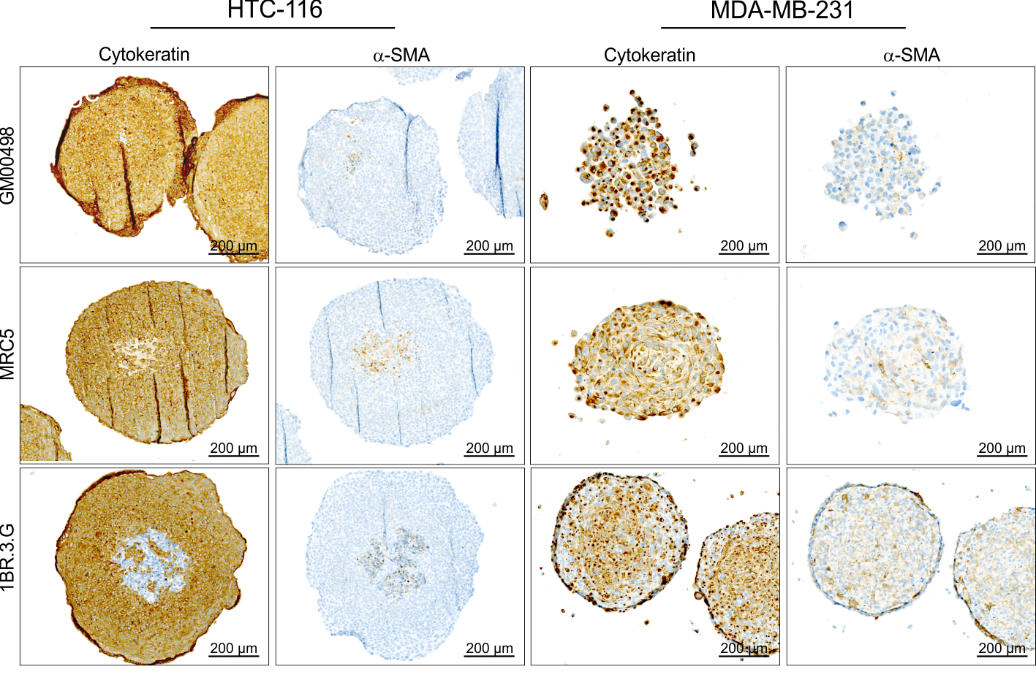


# Supplementary Figure S2

**Expression of CK5 and CDH1 in heterospheroids.** Immunoperoxidase staining of CK5 shows abundant expression in SW480 heterospheroids in contrast to HT29, which are overall CK5 negative. CDH1 is very low in SW480, but highly expressed in HT29 heterospheroids. The 1BR.3.G fibroblasts are negative for both epithelial markers.


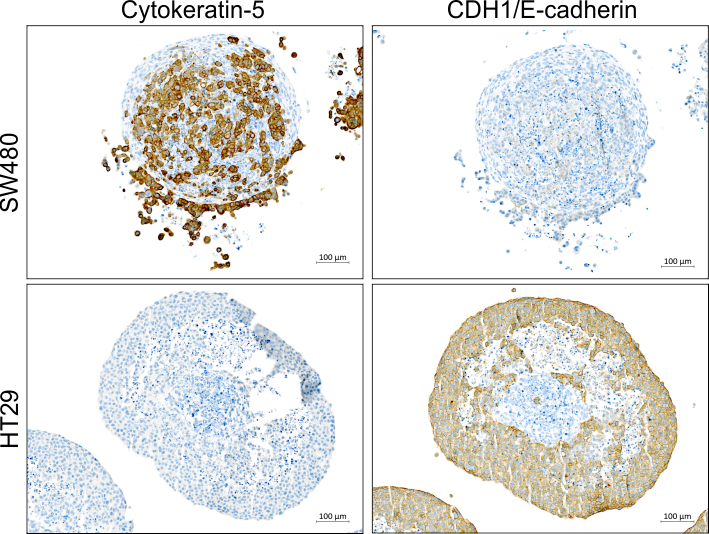


**Supplementary Figure S3**.

Effect of cisplatin on spheroid structures. **A**. HT29 spheroids before and after treatment with low and high level of cisplatin. **B**. Increasing caspase 3/7 levels are indicative of increasing cell death. **** indicates a P-value < 0.0001 using an unpaired T-test.





**Supplementary Figure S4**.

Effect of various drugs on spheroid structures. **A**. HT29 heterospheroids after 72 hrs exposure with selected compounds from NCI library of 166 approved oncology drugs. **B**. Cell viability measured by CellTiter-Glo® viability assay. **C**. Cell death measured by Caspase 3/7 activity assay.


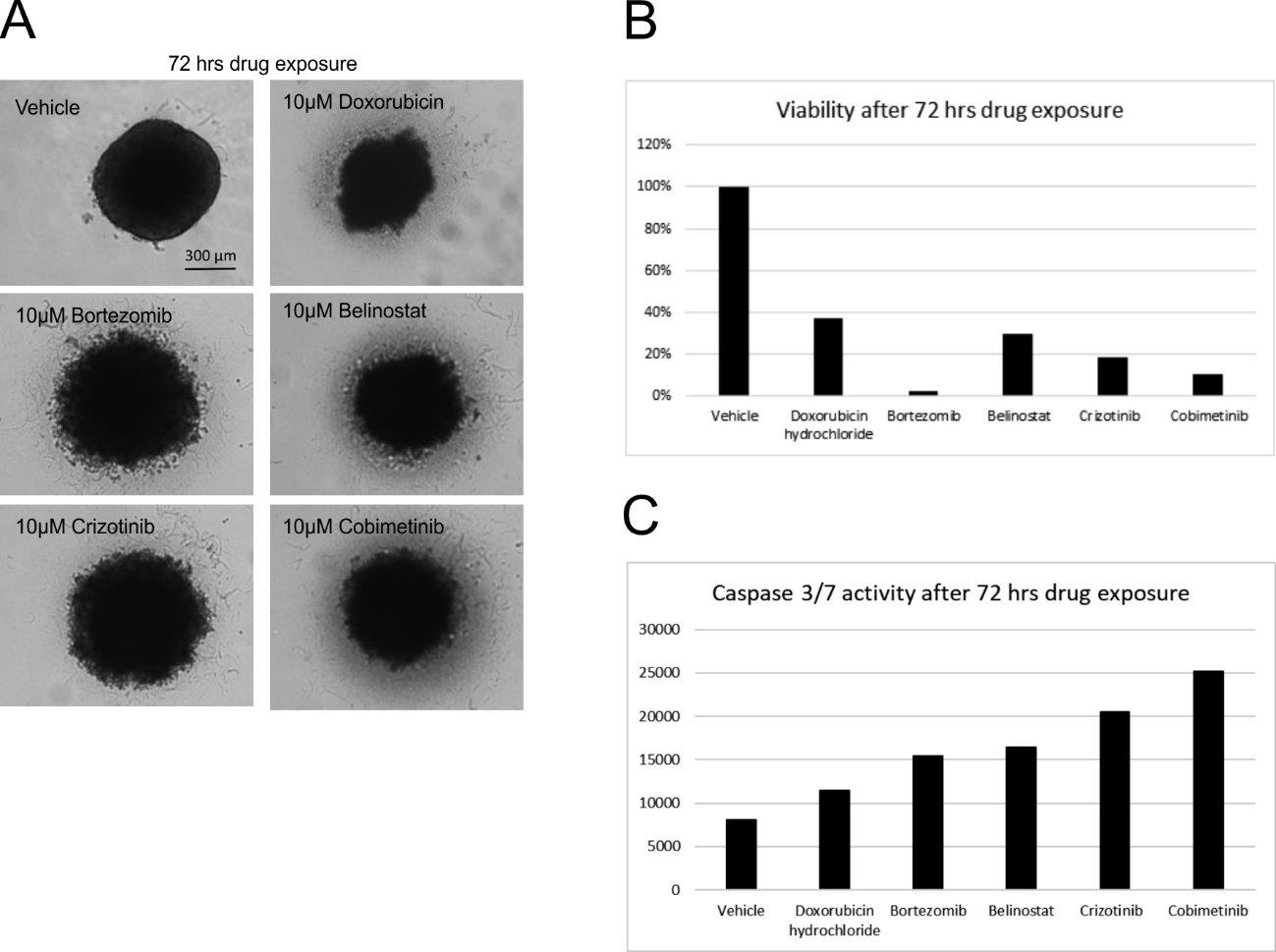

Supplement: Supplementary file 1 [file DataSheet_1.docx]
